# The Unusual Suspects: Esophageal Polyps Causing Dysphagia

**DOI:** 10.14309/crj.0000000000002147

**Published:** 2026-05-22

**Authors:** Mark Ayoub, Ebubekir Daglilar, Bushra Sayeed, Nadia Naumova, Veysel Tahan

**Affiliations:** 1 Division of Gastroenterology, Charleston Area Medical Center, Charleston, WV; 2 Department of Pathology, Anatomy and Laboratory Medicine, West Virginia University, Morgantown, WV; 3 Department of Pathology, West Virginia University, Morgantown, WV

## CASE REPORT

**Figure 1.**
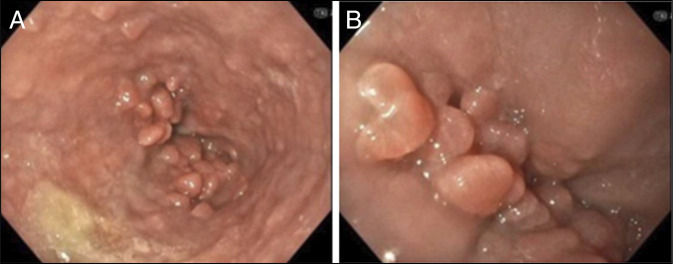
(A and B) Endoscopic images showing multiple 2 mm by 8 mm polyps in the lower third of the esophagus.

A 36-year-old man with Klinefelter syndrome presented with progressively worsening dysphagia and poor oral intake. A computed tomography scan showed mild thickening of the second part of the duodenum with multiple adjacent prominent lymph nodes and fat stranding. Stool *Helicobacter pylori* antigen was negative. An esophagogastroduodenoscopy revealed a cluster of multiple 2 mm by 8 mm polyps in the lower third of the esophagus (Figure 1).

**Figure 2.**
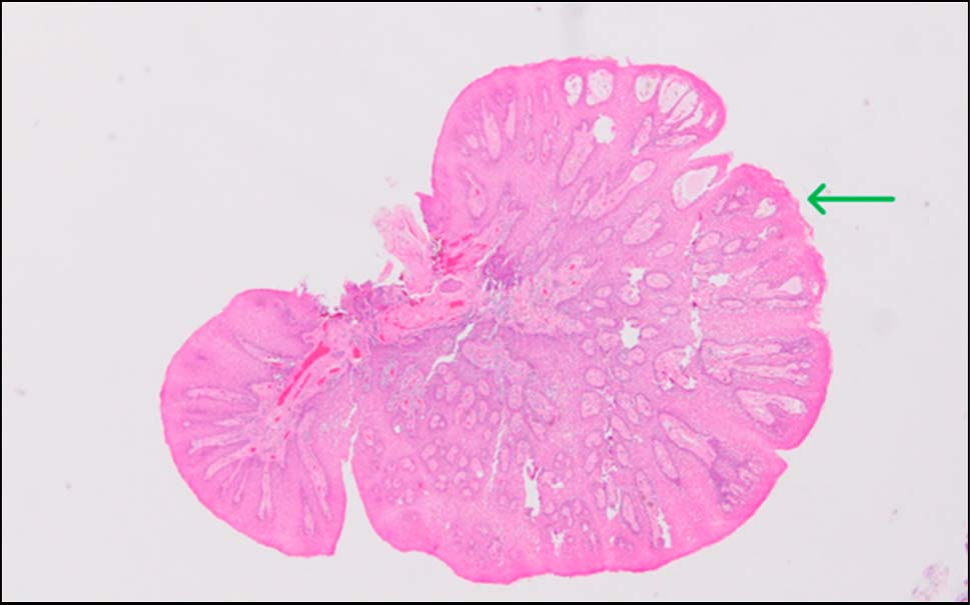
Hematoxylin and eosin stain (4×) shows the esophageal polyps biopsy specimen with polypoid fragments of squamous mucosa with surface erosion and acanthosis (marked by arrow).

Polypectomy using cold snare was sent for pathology. Pathology showed polypoid fragments of squamous mucosa with focal surface erosion and associated acanthosis and reactive epithelial changes, consistent with squamous papilloma (Figure 2).

**Figure 3.**
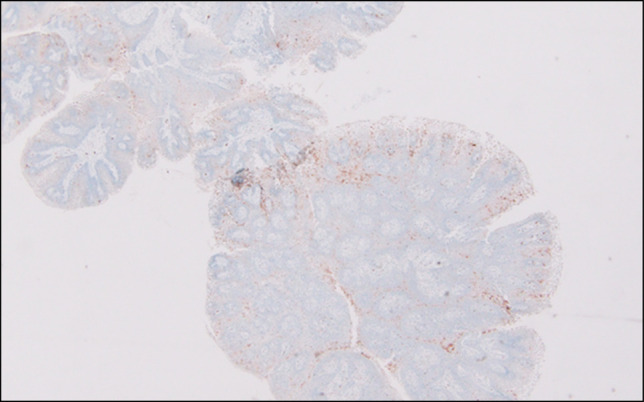
p16 immunostain (4×) is negative for human papillomavirus-mediated process.

Immunohistochemical stain with p16 (surrogate marker for active high-risk human papillomavirus [HPV]), was negative (Figure 3). Our patient's symptoms improved with resection of the papillomas and was able to tolerate regular diet.

Esophageal papillomas are rare with an incidence of 0.1% to 0.6% in patients undergoing esophagogastroduodenoscopy.^1^ Although most esophageal papillomas are asymptomatic, larger or clustered lesions can cause dysphagia, which is typically resolved with endoscopic resection and rarely recurs.^2^ There is evidence that HPV contributes to the development of esophageal papillomas; however, its role in the pathogenesis of esophageal cancer remains unclear.^3,4^ In conclusion, this case emphasizes the uncommon presentation of esophageal papillomas with dysphagia, the role of HPV testing, and the effectiveness of endoscopic resection, which typically results in symptom resolution with a low recurrence rate.

## DISCLOSURES

Author contributions: M. Ayoub: Conceptualization, design, obtaining data/curation, analysis, validation, writing original draft, manuscript review and editing. E. Daglilar: Conceptualization, validation, interpretation, supervision, manuscript review and editing. B. Sayeed and N. Naumova: Obtaining data, analyzing, interpretation, manuscript review and editing. V. Tahan: Conceptualization, design, analyzing, interpretation, supervision, validation, manuscript review and editing. All authors gave final approval of the manuscript to be submitted. M. Ayoub is the article guarantor.

Financial disclosure: None to report.

Informed consent was obtained for this case report.
